# Co-Designing Technology to Reduce Health Disparities and Address New Norms Post–COVID-19: Proposal for a Mixed Methods Community-Based Participatory Research Approach

**DOI:** 10.2196/73927

**Published:** 2025-09-18

**Authors:** Christina K Holub, Konane Martinez, Noe C Crespo, Diane Hoang, Michael Markidis, Tana Lepule

**Affiliations:** 1 Department of Public Health California State University San Marcos San Marcos, CA United States; 2 National Latino Research Center California State University San Marcos San Marcos, CA United States; 3 Institute for Behavioral and Community Health School of Public Health San Diego State University San Diego, CA United States; 4 Independent Senior Software Engineer Consultant San Diego, CA United States; 5 Pacific Islander Collective San Diego San Diego, CA United States

**Keywords:** mobile health, CBPR, community co-design, academic-community partnerships

## Abstract

**Background:**

Everyday life has changed since the COVID-19 pandemic. Existing health disparities among underserved communities have been exacerbated. Latino and Native Hawaiian and Pacific Islander (NHPI) populations disproportionately experience health disparities, even when compared to other minority populations. Both populations have heart disease, cancer, and diabetes as the leading causes of death, and both have high rates of obesity. As we recover from the pandemic, we must consider the intersection of continued health disparities, new social norms and attitudes, and new patterns of health behavior.

**Objective:**

The overarching goal of this project is to reduce health disparities among Latino and NHPI populations, considering new health behavior patterns, social norms, and increased technology use. The research project–specific aims are to (1) conduct key informant interviews and focus groups among Latino and NHPI populations; (2) develop and implement a community health and health behavior survey; and (3) co-design, develop, and test new technology that is meaningful and responsive to community needs and preferences.

**Methods:**

Using community-based participatory research (CBPR) and mixed methods approaches, the interdisciplinary research team will develop new technology based on community insights (key informant interviews, focus groups, and a community health survey). With our community liaisons, we will recruit adult (18+ years old) Latino and NHPI community members from the northern region in San Diego County (ie, Oceanside, San Marcos, and Escondido), largely from culturally related groups and organizations, such as dance schools (hālaus) and churches. Qualitative data will be analyzed using directed content analysis, and quantitative data will be analyzed using descriptive and multivariate statistics. The main outcomes include the identification of community health needs, culturally appropriate interventions, desired modality of intervention strategies, and acceptability of the technology. We expect the new technology to be focused on mobile health (mHealth) smartphone apps. Components will likely include strategies to improve obesity-related health behaviors and mental health.

**Results:**

This study received funding from the National Institute of General Medical Sciences in April 2022 as part of the Support for Research Excellence (SuRE) Program (R16). Key informant interviews and focus groups were completed in July 2023. Community health surveys were completed in August 2024. The development of the beta mHealth app began in September 2024 in partnership with California State University San Marcos (CSUSM) computer science students. Beta testing and evaluation will be completed by December 2025. The qualitative findings, identifying themes for a new mHealth app, were published in June 2025.

**Conclusions:**

A major strength of this study is that it works with the communities of intended impact to directly inform new innovations to promote health behaviors. This study includes unique partnerships and an interdisciplinary team of researchers, students, community members, and consultants/collaborators to inform practices that can impact health disparities among Latino and NHPI populations with technology and strategies that are innovative and effective.

**International Registered Report Identifier (IRRID):**

DERR1-10.2196/73927

## Introduction

### Background

The COVID-19 pandemic has changed life as we know it. Existing health disparities were exacerbated, especially for underserved communities. In response, there has been an increase in government funding opportunities to develop and implement interventions to increase COVID-19 testing and address vaccine hesitancy. As we recover from the pandemic, we must consider the intersection of continued health disparities, new social norms and attitudes, and new patterns of behavior, particularly in how these factors impact health behavior and participation in health behavior interventions. New norms include increases in technology use, and formative research is needed to inform strategies that best capture the needs for reducing health disparities.

Many families, especially with school-aged children, were required to engage in online platforms for work and/or school, pushing our technology and its capabilities to grow faster than originally planned. While the digital divide continued to reveal itself during the pandemic and is still an important concern, the use of smartphones helps to close the gap, as smartphone usage among minority groups is comparable to non-Hispanic Whites, about 85% for adults and over 95% for teens [1,2]. Related to health, Latino/Hispanic people rely heavily on smartphones for health information and are more likely to use health apps, also known as mobile health (mHealth) [3,4]. Culturally tailored mHealth apps offer greater confidence in success [5]. Additionally, based on participant feedback from a pilot study, Native Hawaiian and Pacific Islander (NHPI) participants expressed interest in developing a mobile app that would promote health behavior change and encourage participation in intervention activities. Given the uncertainty of this “new normal,” it is important to partner with communities to better understand how new habits and practices impact their health and health behaviors.

Latino and NHPI populations are some of the fastest-growing minority groups in the United States, with percent changes of 70% and 61%, respectively, between 2000 and 2019 [6]. California is an ideal location to engage with both the Latino and the NHPI communities, since it is home to more than 15 million Hispanic/Latino people and home to over 250,000 NHPI people, more than any other state, even Hawaii. In San Diego County, California, the population is over 30,000 for the NHPI community and about 1 million for Hispanic/Latino people [7]. Latino and NHPI people disproportionately experience disparities in health, even when compared to other minority populations. Both populations have heart disease, cancer, and diabetes as the leading causes of death, and both have high rates of obesity [8]. Over 81% of Hispanic Americans and 76% of NHPI people have overweight or obesity [9]. They also have the highest obesity rate compared to other groups in the United States and are more likely to be inactive, with a smaller percentage of NHPI people meeting federal physical activity (PA) guidelines compared to non-Hispanic White people [9]. Globally, out of the top 10 countries with the highest obesity, 9 were NHPI countries [10]. Diabetes is a persistent health concern among both Latino and NHPI populations. In 2018, close to 20% of NHPI adults had diabetes compared to 12.9% of African American adults, 11.5% of Hispanic adults, and 11.4% of Asian adults [9]. Serious mental illness is also a growing concern, with over 1 in 5 Latino/Hispanic people having a mental illness (about 17% of the population) and being 60% less likely to have received mental health treatment compared to non-Hispanic White people [9,11]. NHPI people also report alarming rates of serious psychological distress, at over 2 million adults between 2015 and 2016 [9]. Based on the 2020 census, 3.5% of NHPI adults experienced a serious mental illness within the past year [12]. These conditions and related health behaviors have been negatively impacted by COVID-19 restrictions, especially PA, sleep, mental health, and overall well-being [13,14]. Improving the health of Latino and NHPI communities is imperative for reducing health disparities. Moreover, working in partnership with communities will provide the best insights into appropriate, culturally relevant strategies.

### Theoretical Framework

There is a growing emphasis on research that is community-based versus “community placed” [15]. This type of research translation highlights community partnership approaches in addressing health and health disparities, especially in diverse cultures. According to the Kellogg Health Scholars Program (Community Track), “CBPR (community-based participatory research) in health is a collaborative approach to research that equitably involves all partners in the research process and recognizes the unique strengths that each brings. CBPR begins with a research topic of importance to the community and has the aim of combining knowledge with action and achieving social change to improve health outcomes and eliminate health disparities“ [16]. Over 15 years ago, the Institute of Medicine recommended CBPR as one of 8 new areas in public health education [17]. Sometimes referred to as “research plus” [18], CBPR not only increases the body of knowledge but also has potential to identify interventions that are sustainable and ready for dissemination because they have been developed with the community at stake [19].

CBPR approaches are iterative in that the process allows for the development of lasting partnerships between researchers and community members. However, critiques of CBPR include the lack of standardization in terminology, measurement, and evaluation [20]. To complement this study’s CBPR approach, we will include concepts from the RE-AIM (Reach, Effectiveness, Adoption, Implementation, and Maintenance) framework, as highlighted by the National Cancer Institute’s Programmatic Area of Implementation Science in the Division of Cancer Control and Population Sciences [21,22]. The RE-AIM framework, which has 5 steps for translating research into practice, has been used to translate research-based interventions into community-based programs [19,23]. RE-AIM can be used for planning, implementing, and evaluating studies. In this study, RE-AIM concepts will guide the type of formative information solicited from Latino and NHPI community members, particularly focusing on “reach,” “effectiveness,” and “adoption” of new technology for health behavior change. Taken together, concepts from the RE-AIM framework, along with the CBPR approach, provide a focused structure for this study.

The overarching goals of this project are to reduce health disparities among Latino and NHPI communities, especially considering new health behavior patterns, social norms, and increased technology use related to the COVID-19 pandemic. Through CBPR, we will partner with both communities to develop new technology—an mHealth smartphone app—to improve the health concerns identified by the communities (eg, obesity-related health behaviors and mental health). This project also focuses on training students who are underrepresented in biomedical research in all phases of the research and innovation development, with an emphasis on training future independent health disparities researchers and scholars. Students from diverse backgrounds and life experiences contribute to a more diverse workforce in the STEM (science, technology, engineering, and mathematics) fields. Beginning with this proposal and the academic-community partnerships established, each stage of work will continue to advance knowledge, practice, and innovative strategies for reducing disparities in Latino and NHPI communities.

### Innovation

This research is novel in its responsiveness to the impact of the pandemic on health behaviors, social norms, practices, and preferences, and ultimately, how these factors influence health in communities that experience great disparities in health outcomes. A key component of this research is the development of a new technology, an mHealth smartphone app based on community insights through the CBPR process and formative research. While there have been some community-informed mHealth interventions [24-27], there is still a need to partner with communities to co-design and develop new technology, especially in partnership with underserved communities.

**Figure 1:**
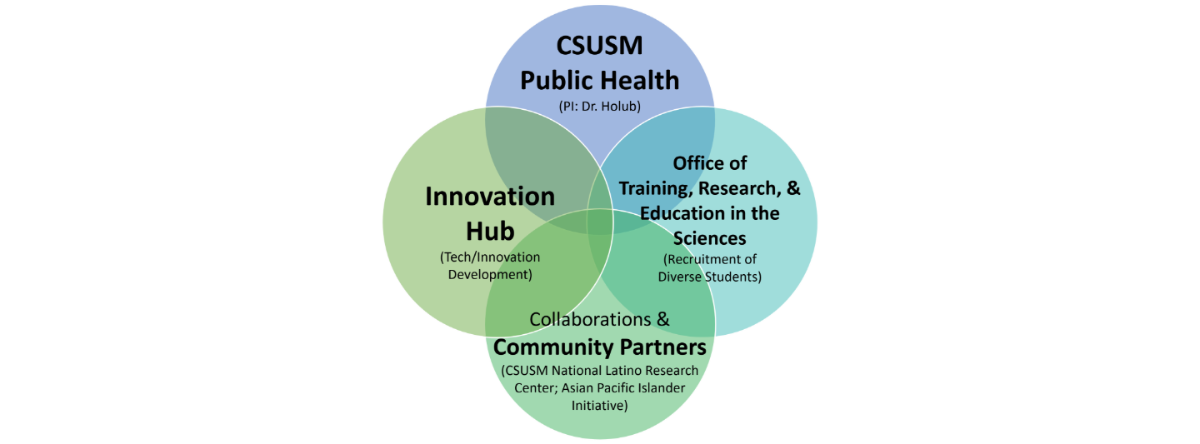
Research team and collaboration framework; CSUSM: California State University San Marcos; PI: principal investigator.

Our collaborative framework includes both community partners and an innovation incubator (Figure 1). Naturally incorporating the services of the California State University San Marcos (CSUSM) Innovation Hub with our senior software engineer consultant, community partners, and collaborators, this research will produce a unique experience in designing new technology for both the academic-community partners and students (underrepresented in biomedical research), highlighting excellence in research and research capacity.

### Preliminary Studies

The first Pacific Islander Community Health (PIC Health) Study began in 2013 by creating an NHPI community advisory board [28]. This formative study was funded by the American Cancer Society’s Health Disparities Program. The PIC Health Study used a mixed methods approach to inform the development of appropriate strategies and culturally sensitive methods for behavior change interventions in the central/southern San Diego NHPI community. Beginning with a qualitative phase, key informant interviews and focus groups provided information for best practices for recruiting NHPI people, introducing research into the community, and cultural concepts for strategies to engage the community. The objectives of the interviews and focus groups were to identify and address potential barriers. Informed by the qualitative phase, a quantitative community health survey was developed to assess the themes identified in the qualitative phase, and other health risk behaviors, including objective measures of PA and obesity. A total of 163 (quantitative) community health surveys were collected from NHPI populations in central/southern San Diego; 31% (n=51) were Native Hawaiian, 20% Samoan (n=33), 47% Chamorro (n=77), and 3% (n=5) Tongan or Tahitian. Responses from the PIC Health survey (also confirmed qualitatively) indicated that interest in health topics was most similar among Native Hawaiians and Samoans, with obesity (mentioned by 65% of Native Hawaiian respondents, 60% of Samoan respondents, and 28% of Chamorro respondents) and diabetes (45% of Native Hawaiians, 52% of Samoans, and 42% of Chamorros) being the most frequently cited concerns (Holub, unpublished data, 2015). That study focused on central/southern San Diego, whereas this proposed research focuses on northern areas of San Diego with a high density of both Latino and NHPI populations, particularly Oceanside, San Marcos, and Escondido.

Based on the original PIC Health Study, a PA community-led intervention among Pacific Islander participants was conducted. This pilot study was funded by the Clinical and Translational Research Institute at the University of California, San Diego; the Academic-Community Partnership Pilot Project (2015); and the Howell Foundation for Women’s Health Research, Community Engagement Initiative (2017).

The primary goal of this pilot study was to be responsive to the formative research of the PIC Health Study, indicating an interest in increasing PA among adult NHPI participants. The pilot study was a 12-week, pretest-posttest intervention study. We recruited 19 NHPI participants aged 18 years and up, among whom 15 (79%) were female and 4 (21%) were male, who did not report more than 150 minutes of MVPA in the last 7 days and who did not have medical conditions that would prohibit moderate-to-vigorous PA. The pilot study findings indicate that at 12 weeks, compared to baseline, the participants (1) had decreased BMIs by an average of 2.3 points (3.6 points for women); (2) were closer to meeting PA guidelines, increasing MVPA minutes per week by about 20 minutes (25 minutes for women), although still under the recommended 150 minutes per week (average: 137 minutes); (3) had increased their fruit and vegetable intake and decreased soda consumption; and (4) moved from “contemplation” to “action” for stages of change related to PA [29]. Key stakeholders (the community health promoter and a fitness consultant) reported that adapting to behavior change may have been difficult for some participants, and future interventions should consider mental health and coping skills strategies to supplement the fitness program.

Feedback from the pilot study led us to conduct focus groups on mental health among the pilot study participants. In 2019 and early 2020, current PA participants (as part of 2 focus groups) discussed mental health. In addition to identifying themes on mental health stigma, motivation, health-seeking behavior, and healthy mindset strategies, the theme most relevant to this proposal is the development of a mobile app for behavior change. Participants indicated that a mobile app that allows tracking of fitness progress may increase retention rates among members. In this study, participants identified the benefits of a mobile fitness app that includes promoting consistent accountability between members and instructors, promoting confidence in individuals to share fitness progress among members, and fitness educational videos to educate beginners and veteran members. One participant mentioned, “I would really like to see a tracking system, to create a consistent accountability. An accountable practice where everybody tracks where they are.”

Based on the literature and preliminary studies, we expect that the proposed project will produce new technology focused on an mHealth smartphone app. Community members will guide the design, but we anticipate that the main components will include resources and strategies to improve obesity-related health behaviors and mental health. With a growing postpandemic need to better understand the intersection of continued health disparities, new social norms and attitudes, new patterns of behavior, and participation in health behavior interventions, the following specific aims were developed.

### Research Project–Specific Aims

#### Specific Aim 1

The first project aim is to conduct key informant interviews and focus groups among the Latino and NHPI participants. Interviews and focus groups will center on gathering information, considering changing norms due to COVID-19, on attitudes, health needs, social norms, cultural beliefs, and preferences that will influence the participants’ ability and willingness to partake in a health behavior intervention. We will conduct between 4 and 6 key informant interviews and 2 focus groups of 6 to 8 participants for each community.

#### Specific Aim 2

The second project aim is to develop and implement a community health and health behavior survey. After completing aim 1, we will develop a survey that includes emerging themes from the interviews and focus groups. Quantitative data will assess social and cultural norms, health behavior knowledge and patterns, use and acceptance of technology, and other health risk behaviors. We will survey 200 Latino and NHPI participants (about 100 from each community).

#### Specific Aim 3

The third project aim is to co-design, develop, and test new technology (beta version), in collaboration with the CSUSM Innovation Hub, that is responsive to community needs and preferences. Based on community feedback and in partnership, we will design and test an app that will help facilitate participation in a health behavior intervention. This app will be designed in a way that can be adapted for each community; for example, content adaptability and language options/controls. A diverse, interdisciplinary team of faculty, students, community members, and consultants will work to create a beta version of the intended product. We will recruit at least 30 participants from each community to test and use the new app. We will evaluate usability, acceptability, and the health behavior outcomes it aims to improve.

### Research Capacity–Specific Aims

#### Specific Aim 1

The first research capacity aim is to enhance the research capacity of students who are underrepresented in biomedical research, especially toward training future independent health disparity researchers and scholars. We will work with the Office for Training, Research, and Education in the Sciences to recruit underrepresented students. We will recruit students from public health, nursing, human development, kinesiology (health science track), business, engineering, and other STEM disciplines. Next, we will evaluate the demographics and number of graduate and undergraduate students trained through this research in each project year, including student outcomes (eg, posters, papers, and graduate school application/acceptance rates).

#### Specific Aim 2

The second research capacity aim is to enhance the research capacity, sustainability, and excellence of CSUSM through meaningful collaborations between the principal investigator and the CSUSM Innovation Hub, the CSUSM National Latino Research Center (NLRC), and other campus collaborators, consultants, community partners, and community advisory boards. To ensure the continued success of these collaborations, we will evaluate attitudes, satisfaction, trust, and perceived impact of the collaboration. A survey will be carried out at the end of years 2 and 4 of the research project.

## Methods

### Overview of Methods

Through the CBPR process and formative research, this study focuses on reducing health disparities for Latino and NHPI communities through the co-design and development of an mHealth smartphone app in collaboration with the CSUSM Innovation Hub. Key informant interviews, focus groups, and a community health survey will be used to collect community-informed formative research. Second, this study highly involves students who are underrepresented in biomedical research with the goal of training future independent health disparity researchers and scholars. This proposal builds on interdisciplinary teams and collaborations to increase research capacity and excellence and address societal needs (Figure 1). Measures to assess underrepresented student involvement (eg, demographics, number, poster presentations, papers, and graduate school application/acceptance rates) and collaborations/partnerships (eg, attitudes, satisfaction, trust, and perceived impact of the collaboration) will be collected.

### Study Research Design and Methods

#### Explanation

This proposal has 3 research project–specific aims and 2 research capacity–specific aims. The research project–specific aims are to (1) conduct key informant interviews and focus groups among Latino and NHPI participants; (2) develop and implement a community health and health behavior survey; and (3) co-design, develop, and test new technology (beta version), in collaboration with the CSUSM Innovation Hub, that is responsive to community needs and preferences. The research capacity–specific aims are to (1) enhance the research capacity of students underrepresented in biomedical research, especially toward training future principal independent health disparity researchers and scholars; and (2) enhance the research capacity, sustainability, and excellence of CSUSM through meaningful collaborations between the principle investigator and the CSUSM Innovation Hub, the CSUSM NLRC, and other campus collaborators, consultants, community partners, and community advisory boards. The RE-AIM Planning Tool will help guide protocol and interview development, ensuring that information is gathered related to concepts of reach, effectiveness, and adoption of new technology [21,22]. The RE-AIM Planning Tool serves as a checklist for key issues to consider when planning and developing an intervention.

#### Participant Recruitment

Latino and NHPI community members from the northern region in San Diego County (ie, Oceanside, San Marcos, and Escondido) will be recruited to participate in either the key informant Interviews (n=4-6 in each community), focus groups (n=6-8 each), survey (n=100 in each community), or product beta testing (n=30 in each community). Working in collaboration with the CSUSM NLRC and the Asian Pacific Islander Initiative, a Latino and NHPI community liaison will assist with participant recruitment. The participant inclusion criteria are as follows: (1) age 18 years or older and (2) identification as Latino, Hispanic, or Native of Samoa, Tonga, Hawaii, Guam, Fiji, or another Pacific Island. The NHPI participants in this study will be recruited from churches, hula “hālaus” (dance schools), and NHPI-related organizations and clubs, with support from a community liaison, who is an NHPI advocate and director of the Asian Pacific Islander Initiative. Our team has existing relationships with these organizations through her previous work. The CSUSM NLRC and collaborators will assist with recruiting Latino participants based on their long-standing relationships in the local area.

Children under the age of 18 will not be included in the proposed study because the intended health benefits are focused on and designed specifically for adult Latino and NHPI participants.

#### Approach and Measures

For the research project–specific aim 1 (interviews and focus groups), the purpose of the key informant interviews is to collect in-depth, qualitative information from people who are familiar with the culture and health patterns of Latino and NHPI communities. We will specifically target community leaders and members who can provide insight into the health concerns of their respective communities. These interviews will be conducted face-to-face by trained student researchers. We will conduct between 4 and 6 key informant interviews. Additionally, we will conduct 2 focus groups with 6 to 8 participants for each community (Latino and NHPI). The focus groups will gather information gained from the interviews and related to health knowledge, cultural beliefs, culturally sensitive methods to conduct research in the community, the acceptability of using new technology like health apps (mHealth), and the identification of barriers and solutions to participating in health behavior interventions. A trained student researcher will conduct and lead the focus group discussion, which will last about 1 hour. A second person will take notes, and the discussion will be audio-taped. Prior to the start of focus groups, a protocol will be developed based on key informant interviews and feedback from the research community boards (Multimedia Appendix 1). All qualitative data will be analyzed for themes to inform the development and design of a quantitative survey.

To address research project–specific aim 2 (community health and health behavior survey), we will collect quantitative data assessing Latino and NHPI cultural norms, health knowledge and screening history, and other risk behaviors, including measures of PA, healthy eating, and obesity. We will survey approximately 100 Latino and 100 NHPI participants. The surveys will be self-administered. The measures listed are not exhaustive, as the qualitative phase of this study will help inform the development of the survey. Questions will include factors in the adoption of new technology, based on the extended unified theory of acceptance and use of technology [30], including performance expectancy, social influence, effort expectancy, facilitating conditions, perceived enjoyment, mobile self-efficacy, satisfaction, and trust [31]. Coping Skills and Cognitive Behavioral Process will be assessed using a 15-item scale [32]. Subjective PA will be assessed with the 16-item Global Physical Activity Questionnaire [33]. Sleep quality and amount will also be assessed. For demographic variables, we will collect information on the participants’ age, gender, marital status, household members (children/adults), employment, household income, and education [34]. Acculturation will be measured using the 8-item Acculturation, Habits, and Interests Multicultural Scale [35]. Perceived religious influences on health behaviors will be measured using a 7-item scale developed by Holt and colleagues [36]. Partner Support will be measured using a 5-item scale [37]. Cultural values around family, respect, and gender roles are fundamental aspects among Latino and NHPI communities. Church/organization food environment around nutrition and healthy eating options during festivals and Sunday gatherings will be assessed. Other risk assessments will include Behavioral Risk Factor Surveillance System items on screening, alcohol use, smoking, and nutrition (National Cancer Institute Fruit and Vegetable Screener). Analyses and results from the qualitative and quantitative data will inform the themes, features, and general design of the new technology.

To address research project–specific aim 3 (co-design/test new technology), we will co-design, develop, and test new technology (beta version), in collaboration with the CSUSM Innovation Hub, that is responsive to community needs and preferences. Based on community feedback from aims 1 and 2 and in partnership, we will test at least one component of the newly developed mHealth smartphone app. This product will be designed in a way that can be adapted for each community (ie, with content adaptability and language options/controls). A diverse, interdisciplinary team of faculty, computer science students, community members, and consultants will work to create a beta version of the intended product. The new technology will be developed by incorporating themes and features into an app development environment. Computer science students will write code to design the app based on community input. We will recruit at least 30 participants from each community to test and use the new mHealth tool. Through feedback surveys and app demonstrations, we will evaluate the usability and acceptability of the new app and the health behavior outcomes it aims to improve (eg, obesity-related health behaviors and mental health). Additionally, we will incorporate an evaluation framework by Maar et al (2017) [38], which outlines an evaluation framework for mHealth interventions in diverse cultural settings. This framework includes the evaluation of major active components of the intervention, technology of the intervention, cultural congruence, task shifting, and unintended consequences [38]. Ultimately, the goal is to create a product for future development (in full) and testing in the project’s next phase (research and funding).

#### Building Research Capacity

To align with this mechanism’s emphasis on research capacity, this project will train undergraduate and graduate students in all aspects of this research, including conducting interviews, focus groups, and surveys, analyzing both qualitative and quantitative data, translating insights from the community to innovation development within an interdisciplinary team, and reporting the findings back to stakeholders and the communities. We will work with the CSUSM Office of Training, Research, and Education in the Sciences to recruit students who are underrepresented in biomedical research. The mission of this office is to provide a supportive multicultural environment for students in the biomedical sciences and related disciplines by sponsoring programs that focus on science education, student support services, research training, and research participation. In this regard, this campus collaboration will support the proposed research study by assisting in the recruitment of underrepresented students.

To demonstrate our commitment to training a diverse group of students who are underrepresented in biomedical research, we intentionally incorporated research capacity–specific aims, which will be measured and evaluated. To assess research capacity–specific aim 1 (training of underrepresented students), at the end of each project year, we will annually evaluate the demographics and number of graduate and undergraduate students engaged in this research project, including other student outcomes (eg, poster presentations, papers, and graduate school application/acceptance rates). We will enhance the research capacity of students from diverse backgrounds, especially toward training future independent health disparity researchers and scholars. We will also work with our campus partners to recruit students from a variety of departments, including public health (graduate students), nursing, human development, biology, kinesiology (health science track), business, and engineering. We anticipate that, across the 4-year project time, at least 20 undergraduate and graduate students will be involved in this study. This type of exposure to health disparities research that is based on the CBPR approach encourages students to pursue similar careers in health disparities research, behavioral sciences, and biomedical research.

Students will be part of interdisciplinary teams, which is tied to research capacity–specific aim 2 (meaningful collaborations). To address this aim, we will enhance the research capacity, sustainability, and excellence of CSUSM through meaningful collaborations between the principal investigator and the CSUSM Innovation Hub, the CSUSM NLRC, the Asian Pacific Islander Initiative, consultants, community partners, and community advisory boards. To ensure the continued success of these collaborations, we will evaluate attitudes, satisfaction, trust, and perceived impact of the collaboration [39]. A survey will be administered to all collaborators at the end of years 2 and 4 of the research project. A unique feature of this proposal is student exposure to innovation development (eg, mHealth tools) through collaboration with the CSUSM Innovation Hub. This exposes students to a diverse, interdisciplinary team spanning public health, anthropology, computer science, and entrepreneurship.

#### Analysis

For qualitative data, interviews from key informants and focus groups will be audio-taped, transcribed, and uploaded into ATLAS.ti (ATLAS.ti Scientific Software Development GmbH), a software used for qualitative coding and analysis. Two individuals, the principal investigator and one trained research assistant, will code and extract themes from the interview/focus group transcripts. Transcripts will be analyzed using directed content analysis to identify and categorize meaningful and detailed information about the participants’ opinions, perceptions, and experiences [40]. Coding strategies will include highlighting passages for emergent themes and a priori categories derived from interviews [41]. Verification of the emergent themes will be conducted through investigator discussions using the developed coding framework. For quantitative data, descriptive statistics will be used to examine each construct in the community survey or beta testing evaluation using SPSS software (IBM Corp). We will use basic bivariate and multivariate analyses to describe the data. Depending on the type of outcome, linear or logistic regression will be used to model the relationship between health behavior outcomes and independent variables (eg, demographics, acculturation, religiosity, stress, etc). Data will be examined by race/ethnicity. We will also examine the usability and acceptability of the mHealth tool, student involvement, and the impact of the collaboration.

#### Project Timeline

Year 1 will be dedicated to the development of the evaluation tools, research team training and recruitment, and completion of key informant interviews and focus groups among the Latino and NHPI communities. Year 2 will focus on community health survey data collection and evaluation of all the formative research. Year 3 will focus on working with the Innovation Hub to co-design and evaluate the new technology (beta testing). Year 4 will be dedicated to finalizing the beta testing/evaluation, interpreting the findings, disseminating results (through conferences, manuscript preparation, and community presentations), and developing a Small Business Technology Transfer (STTR ) application in partnership with our collaborators and Latino and NHPI community advisory boards. Student involvement will be evaluated at the end of each project year, and collaborations will be evaluated at the end of the second and fourth project years (Table 1).

**Table 1:** Study and evaluation timeline.

Activity	Year 1				Year 2				Year 3				Year 4			
	Q1	Q2	Q3	Q4	Q1	Q2	Q3	Q4	Q1	Q2	Q3	Q4	Q1	Q2	Q3	Q4
Protocol and program development	✓	✓														
Develop evaluation tools and material	✓	✓														
Train research team (staff and students)		✓	Ongoing	Ongoing												
Recruit participants (Latino and NHPI^a^)		✓	✓													
Key informant interviews/focus groups (RP^b^ aim 1)			✓	✓												
Community health survey (RP aim 2)					✓	✓	✓									
Evaluation/analysis of formative research				✓			✓	✓								
Work with Innovation Hub to develop a product based on community feedback									✓	✓	✓					
Beta test product (RP aim 3)											✓	✓	✓			
Evaluation/analysis of beta testing														✓	✓	
Evaluate student involvement (RC^c^ aim 1)				✓				✓				✓				✓
Evaluate collaborations (RC aim 2)								✓								✓
Dissemination and manuscript preparation															✓	✓
Development of STTR^d^ grant with CAB^e^															✓	✓

^a^NHPI: Native Hawaiian and Pacific Islander.

^b^RP: research project.

^c^RC: research capacity.

^d^STTR: Small Business Technology Transfer.

^e^CAB: community advisory board.

### Ethical Considerations

This research falls under exemption 2 and exemption 3, as part of the Health and Human Services regulations for the protection of human participants in research [42]. Exemption 2 is met as the first phase of this research involved interviews, focus groups, and surveys among adult Latino and NHPI participants. Both the qualitative and quantitative data will be recorded in a way that the participants cannot be identified. Exemption 3 is met and applied to the last phase of this research, aimed at beta testing new technology. The new technology testing will center on usability, acceptability, and the identified health behavior it aims to change (anticipated to be benign in nature, brief, and harmless). Therefore, informed consent is waived, and participants will be provided with an information sheet (Multimedia Appendix 2).

Data will be collected in a way that cannot identify participants of this study. Research records will be kept confidential to the extent permitted by law. Participants will be identified by an ID code, and personal information from records will not be released without written permission. Participants will not be personally identified in any publication about this study. All sensitive information will be locked/secured or password-protected. Compensation for participants will be in the form of gift cards with the sum of US $30 for interviews, US $20 for focus groups, and US $10 for surveys. Multimedia Appendix 3 provides further explanations of human participant involvement, potential risks, and safety monitoring.

## Results

This study received funding from the National Institute of General Medical Sciences in April 2022 (R16GM145519) as part of the Support for Research Excellence (SuRE) Program (R16). Multimedia Appendix 4 provides the review summary statement. This study also received prior approval by the CSUSM Institutional Review Board in January 2022. Recruitment began in late 2022. Key informant interviews (N=11) and 4 focus groups (N=14) were completed by July 2023. Community health surveys were completed by August 2024 (N=219). The development of the Beta mHealth app began in September 2024 in partnership with CSUSM computer science students who were completing a capstone project in fall 2024. Our project continues to collaborate with computer science students completing a capstone project in spring 2025. Beta testing and evaluation are expected to be completed by December 2025. Findings from the qualitative data (key informant interviews and focus groups), which identify themes for a new mHealth app, have been published [43]. Findings from the mHealth app beta testing are expected to be published by 2027.

## Discussion

### Overview

Given the COVID-19 pandemic’s impact on new health behavior patterns, social norms, and increased use of technology, this proposal lays a strong foundation for collaborative co-design of new technology that advances knowledge, practice, and innovative strategies for reducing health disparities in a postpandemic context. We expect that the proposed project will produce new technology focused on an mHealth smartphone app. Community members will guide the design, but we anticipate that the main components will include resources and strategies to improve obesity-related health behaviors and mental health. A major strength of this study is that it works with the communities of intended impact to directly inform new innovations to promote health behaviors. When co-design and collaboration are priorities, the ability to design culturally tailored mHealth apps is enhanced, offering greater confidence in the success and sustainability of the intervention [5].

While there is little literature on mHealth app development for the NHPI community, there is some information on developing mHealth apps for the Latino community, and some studies have already shown the promise of culturally tailored mHealth apps for Latino/Hispanic people [44,45]. To position this proposal optimally, this work is also guided by a community advisory board and in collaboration with the CSUSM NLRC. This study includes unique partnerships and an interdisciplinary team of researchers, students, community members, and consultants/collaborators to inform practices that can impact health disparities among Latino and NHPI communities, along with technologies and strategies that are innovative and effective.

Additional strengths of this proposed project include the use of the mixed methods design, which facilitates a deeper understanding of community needs and health behaviors. Additionally, the project is guided by strong community engagement principles (ie, CBPR) and cultural relevance. Co-designing the mHealth innovation with the community creates a stronger sense of community ownership and cultural relevance. Some limitations include sample size, the need for expertise in app development beyond computer science students, and geographic constraints, as this study is focused on North County San Diego only.

After completing the objectives of this proposal, future work will focus on refining the new mHealth app and technology based on feedback from the beta version. The integration of artificial intelligence will also be timely, considering the direction of this research and technology development. Once the app is fully developed, a long-term evaluation of its impact on health outcomes will be essential to ensure the effectiveness and sustainability of the mHealth intervention. In the longer term, once the mHealth app is available to the public, the next steps will involve scaling up and addressing broader implications.

### Challenges and Solutions

We recognize the challenges of academic-community partnerships and that the proposed methods may be modified as we obtain additional community input. Any needed precautions related to the pandemic will follow state and county guidelines. The current proposal is informed by preliminary studies, a focus group, and extensive experience working with both the Latino and NHPI communities. To address this potential challenge, we will carefully consider the pace at which we introduce this new study to community members. Ethical considerations also need attention when introducing mHealth technology, including informed consent, social implications, and privacy [46]. We anticipate that with endorsement from community leaders in our advisory board, clear/open communication, flexibility, and with great attention to cultural sensitivity, we can work to overcome these barriers.

## Appendix

Multimedia Appendix 1 Interview and focus group guide.

Multimedia Appendix 2 Information sheet.

Multimedia Appendix 3 Protection of human participants.

Multimedia Appendix 4 Peer review report from ZGM1 RCB-2 (SS) - National Institute of General Medical Sciences Special Emphasis Panel, NIGMS Review of SuRE Applications (National Institutes of Health, USA).

## Abbreviations

CBPR: community-based participatory research
CSUSM: California State University San Marcos
NHPI: Native Hawaiian and Pacific Islander
NLRC: National Latino Research Center
PA: physical activity
PIC Health: Pacific Islander Community Health
RE-AIM: Reach, Effectiveness, Adoption, Implementation, and Maintenance
STEM: science, technology, engineering, and mathematics
STTR: Small Business Technology Transfer
SuRE: Support for Research Excellence
