# Supplementary material for: Co-Designing Technology to Reduce Health Disparities and Address New Norms Post–COVID-19: Proposal for a Mixed Methods Community-Based Participatory Research Approach
Source: JMIR Res Protoc. 2025 Sep 18;14:e73927. doi: 10.2196/73927 (PMC12491890; doi:10.2196/73927)
Supplement: Multimedia Appendix 1 [file resprot_v14i1e73927_app1.pdf]

Date: _________________ Time Start/End: __________/__________

Participant/Group ID: __________ Interviewer Initials: ________

Latino and Pacific Islander Community Health (PIC Health) Study

Key Informant Interview and Focus Group Protocol and Questions

STEP 1. Read the "information sheet" out loud to all potential participant(s).

[Read next section for Focus Groups Only]

Has anyone ever participated in a focus group before? *(Explain that focus groups are being used more and more often in health and human services research.)*

Focus groups are helpful because:

• We learn from you

• We're gathering information, not trying to achieve consensus

• We're seeking to understand the health priorities within your community

Having some ground rules will help us have a good discussion:

(Ask the group to suggest some ground rules. After they brainstorm some, make sure the following are on the list.)

• Everyone is invited to participate.

• Information provided in the focus group must be kept confidential.

• Stay with the group and please don't have side conversations

• Turn off cell phones if possible.

• Have fun.

STEP 2. Ask participant(s) if they have any questions and answer the questions. Remind participant(s) that we are recording the session (audio or video recording) and ask for their permission to record. Once they approve, let the participant(s) know that you will start the interview (or focus group). ****HIT RECORD****

STEP 3. Be sure your research/interview partner is ready to take notes and begin reading the questions and engaging the participant(s).

Date: _________________ Time Start/End: __________/__________

Participant/Group ID: __________ Interviewer Initials: ________

Key Informant Interview and Focus Group Questions

Instructions: Read each question as written, following the given order. Do not finish the participant's sentence. Probing is a good technique to elicit more detailed, adequate responses. The following are examples of probing questions:

- Would you like to elaborate on that?

- Is there anything else you would like to add?

- You mentioned ______, can you tell me more about that?

<u>Section A: Reach and Effectiveness</u>

1. How would you describe your community?

2. What are the best ways to reach (your) Latino/Pacific Islander community?

3. What health concerns or health behaviors do you think most affect the [Latino or Pacific Islander] community?

4. What can we do to address these challenges? (What are some potential intervention strategies to combat those health concerns and to promote health? If participant gives distinct health concerns, ask this question for each one mentioned.)

5. What would make a health intervention successful?

6. Do you think the strategies you mentioned would work for all groups [Latino or Pacific Islander], including those most at risk and having the fewest resources? If no, what can be done to increase the chance of success for these groups?

    a. Notes for interviewer on groups, depending on community:

7. How has COVID-19 affected your daily life? Health? Communication?

8. Has COVID-19 impacted your use of internet and technology?

    a. Technology can include…

9. Do you have home access to the internet?

Date: _________________ Time Start/End: __________/__________

Participant/Group ID: __________ Interviewer Initials: ________

10. Do you use a smartphone? If yes, how comfortable or uncomfortable would you feel about a health app gathering or sending data from your smartphone?

11. Does your phone have a data plan?

12. Do you use your phone or other technology, related to your health or tracking? (Like telehealth video meetings with doctors, smart watches, etc…)

13. In what ways might an app (on your smartphone) be helpful for improving your health?

14. In what ways might an app (on your smartphone) be helpful for improving your community's health?

15. How likely are you to use a mobile health app?

Section B: Adoption and Implementation

Clarify to interviewee: "Now, thinking about the health concerns and potential interventions we talked about…"

1. What do you think will be the greatest barriers to people or organizations adopting a health intervention?

2. How can we overcome these barriers?

3. What cultural beliefs and/or customs should we consider in designing a new health intervention, program, or technology?

4. Do you think your organization would be willing to participate and help recruit participants in a future health intervention or study?

5. What other organizations or community groups do you think would be willing to participate in a future health intervention aimed at [Latinos or Pacific Islanders]?

Date: _________________ Time Start/End: __________/__________

Participant/Group ID: __________ Interviewer Initials: ________

Section C: Demographics

1. What is your age? [Note: if over 89 years old, write: "90+"]

   ______YRS

2. How do you identify...?

   ☐ Woman 1
   ☐ Man 2
   ☐ Transgender 3
   ☐ Non-binary/non-confirming 4
   ☐ Prefer not to respond 5

3. What ethnicity do you identify with?
   ☐Samoan 1
   ☐Tongan 2
   ☐Native Hawaiian 3
   ☐Chamorro 4
   ☐Other Pacific Islander _________________5
   ☐Latino/Hispanic; Specific Group _________________6

4. What languages do you speak?
   ☐ Samoan 1
   ☐ Tongan 2
   ☐ Native Hawaiian 3
   ☐ Chamorro 4
   ☐ Tahitian 5
   ☐ Spanish 6
   ☐ English 7
   ☐ Other 8

Concluding the Interview

Thank the participant for their time. Do not leave in a hurry. Allow a few minutes of winding down conversation. Immediately after leaving, write down any notes about the interview.

Interviewer Notes: Please write down any other notes you think should be included:

____

____

____
