# Supplementary material for: Co-Designing Technology to Reduce Health Disparities and Address New Norms Post–COVID-19: Proposal for a Mixed Methods Community-Based Participatory Research Approach
Source: JMIR Res Protoc. 2025 Sep 18;14:e73927. doi: 10.2196/73927 (PMC12491890; doi:10.2196/73927)
Supplement: Multimedia Appendix 2 [file resprot_v14i1e73927_app2.docx]

**
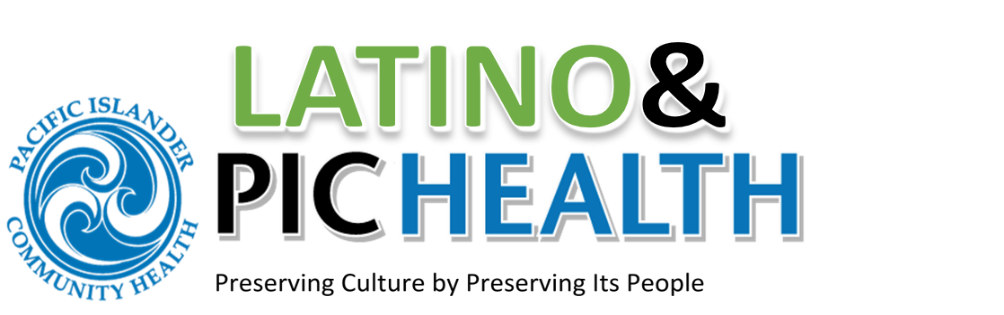
**

**Latino and Pacific Islander Community (PIC) Health Study:**

**Community Health Survey**

Dear Participant,

Greetings! I am Dr. Christina Holub and I am a professor in Department of Public Health at California State University San Marcos. I am conducting a research study to reduce health disparities among Latinos and NHPIs, especially considering new health behavior patterns, social norms, and increased technology use related to the COVID-19 pandemic. The purpose of this form is to inform you about the study.

**Why am I being invited to take part in this study?**

You are invited to take part in this study because you identify as Latino or Native Hawaiian/Pacific Islander and are over 18 years old.

**What will I do if I agree to participate?**

If you agree to participate in the study, you will be asked to complete a community health survey. The survey is anonymous and administered online. Survey time may take about 30 minutes (and no more than 1 hour).

You will receive a $10 giftcard for your time. Instructions for claiming your giftcard will be given at the end of the survey. Survey entries/giftcards are limited and subject to electronic authenticity checks.

**What happens if I say yes, but I change my mind later?**

Your participation in this study is voluntary. You may decline to participate at any time, even after the study has started. If you choose not to participate or to withdraw from the study, there will be no penalty, and you will be able to keep any incentives you have earned up to the point at which you withdraw.

**What are the benefits to me for being in this study?**

There are no direct benefits. Although there may be no direct benefit to you, the possible benefit of your participation is the increase of knowledge of community health and how to address changing norms since the COVID-19 pandemic. This knowledge will help us design new technology to address community needs, such as a mobile health app.

**What happens to the information collected for the study?**

Your responses will be anonymous, since we (researchers) do not have access to any identifiable information through the survey. Your data is coded and assigned a study ID. Your name is not needed nor required to complete the survey, so will not be connected to your data.

The results of this study may be used in reports, presentations, or publications but your name will not be used. Results will only be shared in aggregate form. All data will be secured with password protection, and will contain only coded information, which is entered into the database under an assigned study ID. Only the research team has access to the password-protected database and any study material. Paper material will be kept in a locked office in a locked cabinet. All files, including digital files, will be shredded or erased (digitally) no later than 3 years after the project is completed. Data from this study will not be used in future studies.

**Is there any way being in this study could be bad for me? Is there any risk to me by being in this study? If so, how will these risks be minimized?**

There are minimal risks and inconveniences to participating in this study. This includes feeling uncomfortable answering survey questions about your personal health. The time you spend participating in the study might be considered an inconvenience. However, we will remind all participants that they do not need to answer any question that is uncomfortable. Additionally, we have kept the amount of questions as minimal as possible to reduce the time needed for participation. You are also free to change your mind, at any point, since your participation is voluntary.

**Who should I contact for questions?**

If you have questions about the study, please call me at 760-750-8498 or e-mail me at cholub@csusm.edu with the subject, “Health Study”, if possible. If you have any questions about your rights as a participant in this research or if you feel you have been placed at risk, you can contact the IRB Office at irb@csusm.edu or (760) 750-4029.

PLEASE KEEP THIS INFORMATION SHEET FOR YOUR RECORDS
