# Supplementary material for: Co-Designing Technology to Reduce Health Disparities and Address New Norms Post–COVID-19: Proposal for a Mixed Methods Community-Based Participatory Research Approach
Source: JMIR Res Protoc. 2025 Sep 18;14:e73927. doi: 10.2196/73927 (PMC12491890; doi:10.2196/73927)
Supplement: Multimedia Appendix 3 [file resprot_v14i1e73927_app3.docx]

**Protection of Human Subjects**

**Exemptions:** This research falls under exemption 2 and exemption 3. Exemption 2 is met as the first phase of this research involved interviews, focus groups, and surveys among adult Latino and Native Hawaiian and Pacific Islander (NHPI) participants. Both the qualitative and quantitative data is recorded such that the participants cannot be identified. Exemption 3 is met and applied to the last phase of this research aimed at Beta testing new technology. The new technology testing will center on usability, acceptability, and the identified health behavior it aims to change (anticipated to be benign in nature, brief, and harmless).

**Human Subject Involvement, Characteristics, and Design:** Approximately 250 adult Latino and NHPI community members will be recruited to take part in a key informant interview, focus group, or a community health survey. Approximately 30 adult Latino and NHPI community members will be recruited to take part in Beta testing of new technology, resulting from the formative work. Recruitment will occur in the Northern areas of San Diego, CA, particularly San Marcos, Oceanside, and Escondido. Inclusion criteria include individuals who self-identify as Latino or Hispanic, or Native Hawaiian or Pacific Islander as defined by the U.S. Census Bureau.

The National Latino Research Center (NLRC) at Cal State San Marcos and The Asian Pacific Islander Initiative (led by community advocate, Mr. Tana Lepule) will assist in recruitment of Latino and NHPI participants. NLRC will also assist in coordinating the interviews, focus groups, surveys, and Beta testing among adult Latino participants and Dr. Holub with Mr. Lepule with coordinate the interviews, focus groups, surveys, and Beta testing among NHPI participants.

**Study Procedures, Materials, and Potential Risks:** Data will be collected in a way that cannot identify participants of this study. Key informant interviews will collect in-depth, qualitative information from people who are familiar with the culture and know what is happening in the Latino and Native Hawaiian and Pacific Islander community. We will specifically target community leaders and health professionals who can provide insight into the health their community. Another reason to target community leaders is to gather contacts and connections that could lead to academic-community partnerships. These interviews will be conducted face-to-face by trained student researchers. We will conduct 4-6 key informant interviews, with the possibility of additional interviews resulting from referrals. The focus groups will gather information related to health knowledge, cultural beliefs, culturally sensitive methods to conduct research in the community, the acceptability of using new technology like health apps (mHealth), and the identification of barriers and solutions to participating in health behavior interventions. A trained student researcher will conduct and lead the focus group discussion, which will last about one hour. A second person will take notes and the discussion will be audio-taped. Prior to the start of focus groups, a protocol will be developed based on key informant interviews and feedback from the research community boards. We will also collect quantitative data assessing Latino and NHPI cultural norms, health knowledge and screening history, and other risk behaviors, including measures of physical activity, health eating, and obesity. We will survey approximately 100 Latinos and 100 NHPIs. Surveys will be self-administered.

Potential risks to the participants are minimal. Risks to participants could include discomfort in talking about community health needs related to obesity, diabetes, cancer, or cardiovascular disease. Participation will be entirely voluntary, and participants will be encouraged (and not required nor coerced) to disclose only information that they feel comfortable to share. Focus groups will be audio-recorded and transcribed without any personal identifiers. The facilitator will be trained to recognize such discomfort, interrupt the interview, and offer comfort and reassurance to the participant, if needed.

All documents will be stored in a locked cabinet and all electronic documents or data will be password protected.

**2) Adequacy of Protection Against Risks**

**Informed Consent:** As potential participants are recruited, research staff will review the consent form in detail with the participant and answer all questions before inviting the participant to sign the consent form. The consent form will clearly explain the level of commitment that is necessary to be involved in the research study. A copy of the signed consent form with the Experimental Subjects' Bill of Rights is given to the patient and one copy is kept on file with the PI. The consent protocol is reviewed by California State University San Marcos IRB. A waiver of consent may be sought since the only item connecting the participant to identifiable information would be the consent form itself.

**Protections Against Risk:** Participation will be entirely voluntary, and participants will be encouraged (and not required nor coerced) to disclose only information that they feel comfortable to share. Focus groups will be audio-recorded and transcribed without any personal identifiers. The facilitator will be trained to recognize such discomfort, interrupt the interview, and offer comfort and reassurance to the participant, if needed. In addition, there will be continuous safety surveillance. Participation in the focus groups will involve some loss of privacy due to the interactive nature of the focus groups. However, research records will be kept confidential to the extent permitted by law. Subjects will be identified by an ID code, and personal information from records will not be released without written permission. Participants will not be personally identified in any publication about this study. All sensitive information will be locked/secured or password protected.

**3) Potential Benefits of the Proposed Research to the Subjects and Others**

There is no guaranteed benefit to the subjects who participate in this study. Participants, during survey administration and focus group discussions, may benefit from articulating their concerns and suggestions to improve health interventions to a responsive audience of academic and community researchers. Participants in the Beta testing phase may benefit to the extend the product helps the identified behavior of impact of the product; however, since the focus is on usability and acceptability, there is no anticipated health benefit.

**4) Importance of the Knowledge to be Gained**

Benefits to society include a better understanding of reducing health disparities in the Latino and NHPI populations, feasibility, adherence, and potential effectiveness of a new innovation that would enhance participation in a health behavior intervention. Given the uncertainty of the “new normal” due to COVID-19, it’s important to work with communities to better understand and how new habits and practices impact their health and health behaviors.

**5) Data and Safety Monitoring Plan**

See Data and Safety Monitoring Plan document.

Database Protection: The database will be secured with password protection, and will contain only coded information, which is entered into the database under those identification codes. Electronic communication with outside collaborators involves only unidentifiable information. Reports and annual summaries will not include subject-identifiable material. Each will include the identification code only.

Data Quality and Management: Research staff will review all data collection forms on an ongoing basis for data completeness and accuracy as well as protocol compliance.

Subject Accrual and Compliance: Review of the rate of subject accrual, adherence to inclusion/exclusion criteria will occur weekly during the recruitment phase. Research staff will collect data on compliance to the protocols monthly.

Stopping Rules: This study will be stopped prior to its completion if: 1) study recruitment or retention is too low for the study to provide meaningful results; (2) if the research team finds that the *harm* to study participants outweighs the benefit of the scientific evidence to be accrued by continuing to participate in the study or Beta testing.

The principal investigator and IRB at CSUSM will monitor the data and safety procedures.
