# Supplementary material for: Co-Designing Technology to Reduce Health Disparities and Address New Norms Post–COVID-19: Proposal for a Mixed Methods Community-Based Participatory Research Approach
Source: JMIR Res Protoc. 2025 Sep 18;14:e73927. doi: 10.2196/73927 (PMC12491890; doi:10.2196/73927)
Supplement: Multimedia Appendix 4 [file resprot_v14i1e73927_app4.pdf]

1R16GM145519-01 Holub, Christina

RESUME AND SUMMARY OF DISCUSSION: This new Support for Research Excellence (SuRE) award application seeks support to improve health behaviors in Latino and Native Hawaiian and Pacific Islanders (NHPI) using a community-based approach and app-based intervention. Successful completion of this study will lead to community based technological interventions for communities experiencing health disparities. This is a well written application that brings together a strong interdisciplinary team and multiple contributing entities that make up a strong infrastructure as an ideal environment to support research training for students that will build the research capacity of the Principal Investigator (PI). The PI is well suited for the proposed study and has a demonstrated track record of mentoring students from under-represented groups. The team of collaborators with complementary skills are from diverse ethnicity and race; thus, provides a good model for community engagement. The preliminary qualitative data are strong. Although the rationale presented for the suitability of this application to this mechanism is a major strength, the prior literature to inform the work had not been reviewed well. The participatory research concept was not well implemented, and the evaluation plan was not strong, Lack of shared community decision making in the app development and limited tools undermining innovation were minor weaknesses. The sample size was not well justified and some unclear elements in method and plan were noted. The timeline was considered ambitious. Overall, there was high enthusiasm for the proposed study that is an ideal fit to SuRE award with an intervention tailored to address the health inequities in two culturally different communities.

DESCRIPTION (provided by applicant): Life, as we know it, has changed due to COVID-19. Existing health disparities among underserved communities were exacerbated. Latinos and Native Hawaiians and Pacific Islanders (NHPI) disproportionately experience disparities in health, even when compared to other minority populations. Both populations have heart disease, cancer, and diabetes as leading causes of death; and both Latinos and NHPIs have high rates of obesity. As we begin to recover from the pandemic, we must consider the intersection of continued health disparities, new social norms and attitudes, and new patterns of health behavior. The overarching goal of this project is to reduce health disparities among Latinos and Native NHPIs, considering new health behavior patterns, social norms, and increased technology use related to COVID-19. The research project specific aims are to: (1) conduct key informant interviews and focus groups among Latinos and NHPIs, (2) develop and implement a community health and health behavior survey, and (3) based on community feedback [from aims 1 and 2], co-design, develop and test new technology, in collaboration with the CSUSM Innovation Hub, that is meaningful and responsive to community needs and preferences. Additionally, with commitment to training students underrepresented in biomedical research, we were purposeful in outlining research capacity specific aims. They are, to: (1) enhance the research capacity of students underrepresented in biomedical research, especially towards training future independent health disparity researchers and scholars, and (2) enhance the research capacity, sustainability, and excellence of CSUSM through meaningful collaborations between the Principle Investigator and the CSUSM Innovation Hub, OTRES (for recruitment of underrepresented students), and other collaborators and community partners. We will use a Community-based Participatory Research (CBPR) approach and formative research to co-design, develop and test new technology, an mHealth smartphone application, based on community insights. Components will likely include strategies to improve obesity- related health behaviors and mental health. Research capacity: We will evaluate the demographics and number of students involved in research, including student outcomes (e.g., posters, papers, and graduate school application/acceptance rates). We will also evaluate attitudes, satisfaction, trust, and perceived impact of the collaboration. The proposed research lays the foundation for purposeful and progressive funding (i.e., this R16, STTR, R21, R01) that will advance the science and innovation in health disparities research.

PUBLIC HEALTH RELEVANCE: The goal of this project is to reduce health disparities among Latinos and Native Hawaiians and Pacific Islanders (NHPI), especially considering new health behavior patterns, social norms, and increased technology use related to the COVID-19 pandemic. Through Community-

based Participatory Research (CBPR), we will partner with Latino and NHPI communities to co-design and develop new technology, an mHealth application for smartphones, to improve the health concerns identified by the communities (e.g., obesity-related health behaviors and mental health). This project also focuses on training students from underrepresented in biomedical research in all phases of the research and innovation development, with an emphasis on training future, independent health disparities researchers and scholars.

CRITIQUES: *The written critiques of individual reviewers were prepared prior to the review meeting and may not have been revised/edited by the reviewers after the meeting. The "Resume and Summary of Discussion" above summarizes the final opinions of the review panel.*

CRITIQUE 1

Significance: 2
Investigator(s): 2
Innovation: 2
Approach: 3
Environment: 1

Overall Impact:

The proposed project aims to improve health behaviors among Latino and Native Hawaiian and Pacific Islander communities through an app-based intervention developed through an iterative mixed method research process. Specifically, the research team aims to first conduct qualitative research with about 20 Latino and NHPI community members, then develop a survey based on themes taken from the qualitative research and administer it to 200 Latino and NHPI community members, and finally to use the re-AIM framework to translate research results into an app-based health behavior intervention that will be tested by at least 60 participants. The greatest strength of the project is its fit with the SURE grant mechanism; specifically, the proposed project brings together a strong interdisciplinary and cross-sector team (namely the university's Innovation Hub, its Office for Training, Research and Education in the Sciences (OTRES), and the National Latino Research Center (NLRC)) that will not only support research training for students but will also build the research capacity of the PI and CSUSM as a whole. The environment of CSUSM is ideal; the relatively young university primarily serves undergrads, they have a high URM student body, they meet the criteria of less than $6 million in research funding, and lots of programs and supports for undergrads in research. The PI is uniquely well-equipped to lead the project, as she not only has as strong research record but also invests considerable time and energy into mentoring students, particularly students from underrepresented backgrounds. Enthusiasm for the project is dampened by the lack of shared leadership with community partners in research design and implementation, despite the framing as a Community-based participatory research (CBPR) project. The primary deliverable, an app-based intervention to improve health behavior, will be informed by the community voice collected in the first two phases of the project, but no specific plan for shared decision-making is described. A community partner who heads a NHPI community initiative is named as part of the research team and this person provides a letter of support stating general enthusiasm for the project, but he is not a funded member of the team and his role appears to be limited to helping to recruit research subjects. Overall, one of the proposal's primary rationales is the need to "partner" with NHPI communities in order to develop culturally appropriate health behavior interventions, but the extent of community partnership appears to be recruiting research participants whose perspectives will inform the mixed methods research process.

1. Significance:

Strengths

- Prior research is rigorous and provides relevant evidence laying the foundation for the proposed research activities (particularly in the realm of culturally tailored interventions and technology for health behavior interventions).
- Successful completion of the project will lead to a community-informed app that is suited specifically for two communities that experience health inequities.
- CSUSM is a primarily undergraduate research institution and has many existing structures that make it well poised to support students who engage in the proposed research activities.
- The proposed project brings together a strong interdisciplinary and cross-sector team that will not only support research training for students, but will also build the research capacity of the PI and CSUSM as a whole (namely the CSUSM Innovation Hub, Office for Training, Research and Education in the Sciences (OTRES), the National Latino Research Center (NLRC), and community advisory boards).
- Project will partially fund 7 students per year (3 graduate and 4 undergraduate).

Weaknesses

- Despite describing CBPR as an approach that shares decision-making with community members throughout the research process, the proposal does not describe how community members share decision-making but only how they are participants in a research process. Consequently, the benefits to research relevance and impact are truncated.

2. Investigator(s):

Strengths

- PI has a demonstrated track record of mentoring and supervising student research, including 110 capstones and thesis in just five years at CSUSM. At least half of her student mentorship focuses on students from underrepresented backgrounds.
- PI has a strong record of community-focused health behavior research that sets the stage for the proposed research activities.
- Consultation from Martinez as Director of the National Latino Research Center is an excellent interdisciplinary collaboration that strengthens the proposal as a whole.
- Software engineer as part of the team is necessary and appropriate for planned activities.
- Research team diverse in race, ethnicity, gender, and discipline, which provides valuable model to student mentees of diverse team science.

Weaknesses

- The only budgeted community collaboration is $600 per year for each of 2 community health workers, making it clear that community partners are not actually part of the research team.

3. Innovation:

Strengths

- Although behavior change apps are prolific, ones informed by iterative research processes that allow for cultural specificity are rare. The creation of this app has the potential to reach two underserved communities in new and innovative ways.

Weaknesses

4. Approach:

Strengths

- The mixed method approach to tech-based intervention development holds promise for culturally tailored deliverable that are accessible and applicable to Latino and NHPI communities.
- Use of Re-AIM planning tool for intervention development provides appropriate structure to that process.

Weaknesses

- Although the project is described as rooted in a CBPR approach, community partners are not represented in the budget (with the exception of 2 community health workers receiving $600 per year for 2 years). The lack of support for community partners undermines the confidence that the research will reap the benefit of shared decision-making with community stakeholders.

5. Environment:

Strengths

- Well-presented rationale from CSUSM about why they are the ideal setting for this grant mechanism (lots of undergrads, high URM student body, less than $6 million in research funding, lots of programs and supports for undergrads in research).
- The proposal takes advantages of several existing supports at CSUSM to not only strengthen the research, but also student training and experience (e.g., National Latino Research Center, OTRAS, Innovation Hub).

Weaknesses

Protections for Human Subjects:

Acceptable Risks and/or Adequate Protections.

- No issues noted.

Data and Safety Monitoring Plan (Applicable for Clinical Trials Only):

Not Applicable (No Clinical Trials)

Inclusion of Women, Minorities and Individuals Across the Lifespan (Applicable Only for Human Subjects research and not IRB Exemption #4)

- Sex/Gender: Distribution justified scientifically.
- Race/Ethnicity: Distribution justified scientifically.
- For NIH-Defined Phase III trials, Plans for valid design and analysis: Not applicable.
- Inclusion/Exclusion Based on Age: Distribution justified scientifically.
- focus on Latino and NHPI communities is a strength.

Vertebrate Animals:

Not Applicable (No Vertebrate Animals)

Biohazards:

Not Applicable (No Biohazards)

Resubmissions:

Renewal:

Select Agent Research:
Not Applicable (No Select Agents)

Resource Sharing Plans:
Not Applicable (No Relevant Resources)

Authentication of Key Biological and/or Chemical Resources:
Not Applicable (No Relevant Resources)

Budget and Period of Support:
Recommend as Requested.

CRITIQUE 2

Significance: 2
Investigator(s): 2
Innovation: 5
Approach: 3
Environment: 1

Overall Impact:
This is a strong, well-written proposal that will develop and test a beta version of a mHealth application to address a health condition prioritized by Latino/Hispanic & NHPI communities and that is developed in a manner that engages the communities and follows CBPR principles. Both populations suffer from health disparities that have been exacerbated by the COVID pandemic. The research approach is thoughtful, with formative research preceding the app development. Community input and an understanding of the post-COVID ‘new normal’ will be sought via key informant interviews and focus groups. The qualitative research will inform a qualitative survey to be delivered to 200 members of the target populations. Data from both sources will be used to develop the intervention/app. The design of the current proposal parallels that of a recent, successful project led by the PI, which increases the likelihood of success for the proposed research. The PI and collaborators have strong ties to the target communities, and the PI is supported by an institutional climate that values community engaged and inclusion of minorities. The research team is strong, with a longstanding commitment to community-engaged research. The PI has a strong history of working with students, and the application provides a thoughtful student development plan. Weaknesses of the proposal though generally minor include a weak review of prior research, lack of a compelling reason for targeting both populations in this proposal, lack of clarity around engagement of community members in the app development, and lack of clarity about whether the research team has collaborated before this proposal.

1. Significance:

Strengths

- Latino/Hispanic and NHPI communities experience longstanding health disparities, which have been exacerbated by the COVID pandemic.
- Proposed project has strong promise to develop a mHealth approach to support health improvements in these populations.
- Project builds on PI strengths and extends her research area in a thoughtful way, and with complementary collaborators within and outside her institution, and is likely to enhance her research productivity.
- Student research experience and evaluation plan is well-constructed.

Weaknesses

- Although the investigators make a strong case for needing new health interventions that account for the post-COVID ‘new normal,’ there is little review of prior research (aside from participant feedback from PI’s recent work) that informs the current research

2. Investigator(s):

Strengths

- PI has prior experience with both target populations, although most recent work (& strong community partnerships) with NHPIs.
- PI has strong track record with community-engaged research and is expected to provide a strong CBPR introduction to undergrads and graduate students.
- PI has strong record of mentoring students, including co-publishing with students.
- Co-Is provide complementary experience, including additional interventional expertise and relationships to Latino/Hispanic communities, but like PI, are grounded in community-engaged research.

Weaknesses

- Unclear whether the research team has any history of collaboration, which would strengthen the application.

3. Innovation:

Strengths

Weaknesses

- Components of project – mHealth-based behavioral intervention & CBPR – aren’t really innovative even though well-integrated & appropriated.

4. Approach:

Strengths

- Strong CBPR approach that thoughtfully integrates and responds to community perspectives.
- Design of current proposal mimics that of another recent successful project led by the PI.
- Approach includes aims and corresponding evaluations that address both the research project and the process of enhancing research capacity.
- Student involvement plan well-developed, with high likelihood of success..

Weaknesses

- Unclear how current research addresses weaknesses in prior research, aside from PI’s recent work

- Application would be strengthened by more detail around how community members will participate in the research, e.g., including community members in the budget, especially for CAB duties and work with the research team around Aim 3.
- Additional literature to support choice of 2 focus groups per target population (do you expect saturation so quickly?) would strengthen the application.
- Not clear that inclusion of both target populations (Latino & NHPI) will benefit the research, other than helping to extend PI research within Latino populations. Is it expected that both populations will settle on similar health priorities and express similar preferences for intervention design/components? What will happen if these differ?

5. Environment:

Strengths

- CSUSM is classified as a “community-engaged” university & a Hispanic Serving Institution.
- CSUSM has a large proportion of students from underrepresented minorities.
- Institutional infrastructure that will support research – e.g., National Latino Research center; OTRES to help with student recruitment; Office of Graduate Studies and Research (OGSR) provides travel funds for students to present their research outside the university.
- Strong letters of support from community.
- PI appears to have adequate institutional support to conduct the proposed research.

Weaknesses

- None noted.

Protections for Human Subjects:

Acceptable Risks and/or Adequate Protections.

Data and Safety Monitoring Plan (Applicable for Clinical Trials Only):

Acceptable.

Inclusion of Women, Minorities and Individuals Across the Lifespan (Applicable Only for Human Subjects research and not IRB Exemption #4)

- Sex/Gender: Distribution justified scientifically.
- Race/Ethnicity: Distribution justified scientifically.
- For NIH-Defined Phase III trials, Plans for valid design and analysis: Not applicable.
- Inclusion/Exclusion Based on Age: Distribution justified scientifically.

Vertebrate Animals:

Not Applicable (No Vertebrate Animals)

Biohazards:

Not Applicable (No Biohazards)

Resubmissions:

Renewal:

Select Agent Research:

Not Applicable (No Select Agents)

Resource Sharing Plans:

Unacceptable.

- None provided.

Authentication of Key Biological and/or Chemical Resources:

Not Applicable (No Relevant Resources)

Budget and Period of Support:

Recommend as Requested

CRITIQUE 3

Significance: 1
Investigator(s): 1
Innovation: 3
Approach: 4
Environment: 1

Overall Impact:

This is a new proposal from an experienced applicant. The proposal describes to address health disparities in Latino and Native Hawaiian and Pacific Islanders (NHPI) using a community-based approach and technology. The proposal seeks to conduct key formative data collection to identify new needs and barriers related to COVID-19/post-pandemic patterns to then develop and test new technology to address needs. The proposal also highlights a student training component.

The project aims include qualitative methods using interviews and focus groups to inform the development of health behavior intervention; conducing a community health needs assessment related to health behavior and acceptance of technology; and lastly, to develop and test new technology to address needs.

This is a solid proposal with scientific merit however the lack of a conceptual framework, detail about procedures, and evaluation somewhat detract from the proposal.

1. Significance:

Strengths

- Latinos and NHPIs are among the fastest growing minority populations.
- COVID-19 pandemic has led to a shift in norms.
- Health disparities have been exacerbated by COVID-19.
- Technology use and uptake as changed due to COVID-19.
- While the digital divide exists, usage patterns also differ between groups.

- Mental health and weight-related health problems are prevalent across groups.

Weaknesses

- None noted.

2. Investigator(s):

Strengths

- Doctoral Degree in Health Behavior.
- The candidate has strong foundation to complete this work.
- The candidate has a strong publication record (4 articles/3 first authored).
- Has received some scholarships and honors.
- NIH LRP recipient.
- Solid mentorship record.
- Team is experienced in behavioral science, health behavior, community-based approaches, anthropology, immigrant health, and Latino Health and health disparities.

Weaknesses

- None noted.

3. Innovation:

Strengths

- Responsiveness to impact on the pandemic on health behaviors and outcomes.
- Development of a community-informed mHealth intervention.

Weaknesses

- Details about components of mHealth tool are limited, assessing innovation is difficult.

4. Approach:

Strengths

- The research project aims include qualitative methods using 4-6 interviews and two focus groups to inform the development of health behavior intervention on; conducing a community health needs assessment related to health behavior and acceptance of technology n=200; and lastly, to develop and test new technology to address needs.
- The proposal leverages key relationships and supports to support the planned work.
- Research capacity aims include training opportunities for underrepresented students.

Weaknesses

- Lacks detailed conceptual framework and evaluation plan.
- The interview and focus group samples sizes seem small. More justification is needed to support how these samples sizes will be sufficient.
- Aim 2 of the Research Capacity Aim is unclear, is this aim simply an evaluation of the training program or opportunities offered?
- The mHealth technology development process is under explained.
- Evaluation and testing of the app is underdeveloped.

5. Environment:

Strengths

- The environment of California State University San Marcos seems to be robust and supportive for completing the project objectives.
- The applicant has sought relationships with three key centers on their campus including an Innovation Hub, National Latino Research Center, and their campus training office.
- Supportive letters were provided demonstrating the university commitment to faculty research and Dr. Holub's submission specifically.

Weaknesses

- The timeline is ambitious, but the applicant appears to have sufficient support to complete the proposed project activities.

Protections for Human Subjects:

Acceptable Risks and/or Adequate Protections.

- Acceptable.

Data and Safety Monitoring Plan (Applicable for Clinical Trials Only):

- Acceptable.

Inclusion of Women, Minorities and Individuals Across the Lifespan (Applicable Only for Human Subjects research and not IRB Exemption #4)

- Sex/Gender: Distribution justified scientifically.
- Race/Ethnicity: Distribution justified scientifically.
- For NIH-Defined Phase III trials, Plans for valid design and analysis: Not applicable.
- Inclusion/Exclusion Based on Age: Distribution justified scientifically.
  - Acceptable.

Vertebrate Animals:

Not Applicable (No Vertebrate Animals)

Biohazards:

Not Applicable (No Biohazards)

Resubmissions:

Renewal:

Select Agent Research:

Not Applicable (No Select Agents)

Resource Sharing Plans:

Unacceptable.

- Not provided.

Authentication of Key Biological and/or Chemical Resources:

Not Applicable (No Relevant Resources)

Budget and Period of Support:

Recommend as Requested

THE FOLLOWING SECTIONS WERE PREPARED BY THE SCIENTIFIC REVIEW OFFICER TO SUMMARIZE THE OUTCOME OF DISCUSSIONS OF THE REVIEW COMMITTEE, OR REVIEWERS' WRITTEN CRITIQUES, ON THE FOLLOWING ISSUES:

PROTECTION OF HUMAN SUBJECTS: ACCEPTABLE

INCLUSION OF WOMEN PLAN: ACCEPTABLE

INCLUSION OF MINORITIES PLAN: ACCEPTABLE

INCLUSION ACROSS THE LIFESPAN: ACCEPTABLE

COMMITTEE BUDGET RECOMMENDATIONS: The budget was recommended as requested.

Footnotes for 1 R16 GM145519-01; PI Name: Holub, Christina Kim

NIH has modified its policy regarding the receipt of resubmissions (amended applications).See Guide Notice NOT-OD-18-197 at https://grants.nih.gov/grants/guide/notice-files/NOT-OD-18-197.html. The impact/priority score is calculated after discussion of an application by averaging the overall scores (1-9) given by all voting reviewers on the committee and multiplying by 10. The criterion scores are submitted prior to the meeting by the individual reviewers assigned to an application, and are not discussed specifically at the review meeting or calculated into the overall impact score. Some applications also receive a percentile ranking. For details on the review process, see http://grants.nih.gov/grants/peer_review_process.htm#scoring.

MEETING ROSTER

The roster for this review meeting is displayed as an aggregated roster that includes reviewers from multiple GM Special Emphasis Panels of the NIGMS Review of SuRE Applications for the 2022/01 council round.
This roster for GM is available at:
http://public.era.nih.gov/pubroster/Reports?DOCTYPE=SEP&DESFORMAT=PDF&AGENDA_SEQ_NUM_P=430606
